# Metabolically Active Brown Adipose Tissue Is Found in Adult Subjects with Type 1 Diabetes

**DOI:** 10.3390/ijms20235827

**Published:** 2019-11-20

**Authors:** Olof Eriksson, Ram Kumar Selvaraju, Marie Berglund, Daniel Espes

**Affiliations:** 1 Science for Life Laboratory, Department of Medicinal Chemistry, Division of Molecular Imaging, Uppsala University, 75123 Uppsala, Sweden; olof.eriksson@ilk.uu.se (O.E.); ramkumar.selvaraju@ilk.uu.se (R.K.S.); 2 Turku PET Centre, Åbo Akademi, 20520 Turku, Finland; 3 Department of Public Health and Caring Sciences, Uppsala University, 75237 Uppsala, Sweden; marie.berglund@neuro.uu.se; 4 Department of Medical Sciences, Uppsala University, 75185 Uppsala, Sweden; 5 Department of Medical Cell Biology, Uppsala University, 75123 Uppsala, Sweden

**Keywords:** type 1 diabetes, brown adipose tissue, positron emission tomography, metabolic control

## Abstract

Type 1 diabetes (T1D) is characterized by the loss of insulin-producing cells and hence insulin secretion and metabolic control. In addition to insulin, there are a number of hormones and cytokines that influence metabolism, and many of these can be secreted from brown adipose tissue (BAT). However, the presence and activity of BAT in T1D have not been studied, despite the fact that preclinical studies have shown that transplantation of BAT in mouse models of T1D can restore metabolic control. The metabolic activity of BAT, white adipose tissue (WAT), and skeletal muscle was investigated in patients with T1D (*n* = 11) by 2-deoxy-2-(^18^F)fluoro-D-glucose PET/CT after cold stimulation. Functional BAT was detected in 4 out of 11 individuals with T1D with a prevalence of 36%. The glucose utilization rate in the supraclavicular BAT regions ranged from 0.75–38.7 µmol × min^−1^ × 100 g^−1^. The glucose utilization per gram tissue was higher in BAT when compared with both WAT (*p* = 0.049) and skeletal muscle (*p* = 0.039). However, no correlation between BAT activity and metabolic control or insulin requirements was found. In conclusion, for the first time, cold-induced BAT was detected in patients with T1D with a wide range in metabolic activity. Contrary to findings in animal models, the metabolic activity of BAT had negligible impact on insulin requirements or metabolic control in T1D under normal physiological conditions.

## 1. Introduction

Type 1 diabetes (T1D) is characterized by a progressive loss of beta cells and thereby insulin secretion, resulting in hyperglycaemia. Since the discovery of insulin by Banting and Best in 1921, exogenous insulin has been the cornerstone of T1D treatment. In fact, since then, no fundamentally new drug treatments have been clinically established for the treatment of T1D. However, apart from insulin there are a number of hormones and cytokines that influence glucose homeostasis. Cytokines have traditionally been studied for their inflammatory effects, but have in recent years also attracted increasing interest due to their effects on metabolism. For instance, IL-6 has a complex role in metabolism and has been shown to increase insulin sensitivity [1,2], but has also been linked to insulin resistance [3]. Many of the cytokines and hormones that affect glucose homeostasis have been found to be secreted from brown adipose tissue (BAT). Notably, it has been shown that transplantation of BAT to T1D mice restores normoglycaemia without increasing insulin levels [4,5]. In studies of rodents rendered diabetic by the toxic substance streptozotocin, it has been shown that BAT is atrophied and that metabolic activity is reduced [6]. Thus, BAT seems to play a crucial role in glucose homeostatasis in T1D models in rodents, potentially both through hormone regulation and by acting as a glucose “sink” due to its high glucose utilization. Importantly, as the PET technique is fully quantifiable, it is possible to accurately assess the numerical glucose utilization per gram in different tissues, including BAT.

Functional BAT was only recently confirmed in adult humans through hybrid positron emission tomography (PET) and computed tomography (CT) studies [7,8]. The metabolic activity of BAT is physiologically stimulated by cold exposure, and can thus be readily be detected through increased uptake of the radiolabeled glucose analogue 2-deoxy-2-(^18^F)fluoro-D-glucose (^18^F-FDG). Interestingly, the metabolic activity of BAT is blunted in patients with obesity and type 2 diabetes [9,10]. Thus, metabolically active BAT seems to also be linked with the glucose metabolism in humans.

There is also an increasing interest in the hormones and cytokines secreted by BAT (“batokines”) and their beneficial impact on whole body metabolism [11]. Great effort has been expended on developing drugs that mimic the effects of batokines and/or increase the metabolic activity of BAT. We recently found that the widely debated myokine irisin, which has been proposed to have browning effects on white adipose tissue (WAT), is increased in individuals with T1D [12].

Despite these interesting preclinical and clinical data on the role of BAT in metabolism in metabolic disease, there have been no reports on the activity and occurrence of BAT in T1D.

In the present study, we have investigated metabolic activity, measured as glucose utilization rate, in three different tissue compartments, BAT, WAT, and skeletal muscle by ^18^F-FDG PET/CT in response to cold stimulation in 11 individuals with long-standing T1D. The primary endpoint was to evaluate the correlation between the glucose utilization in BAT and insulin requirements in individuals with established T1D. All participants were characterized regarding metabolic control, insulin requirements, body composition, and hormonal status in order to study potential correlations to metabolic activity in different tissue compartments, with emphasis on BAT.

## 2. Results

**Figure 1:**
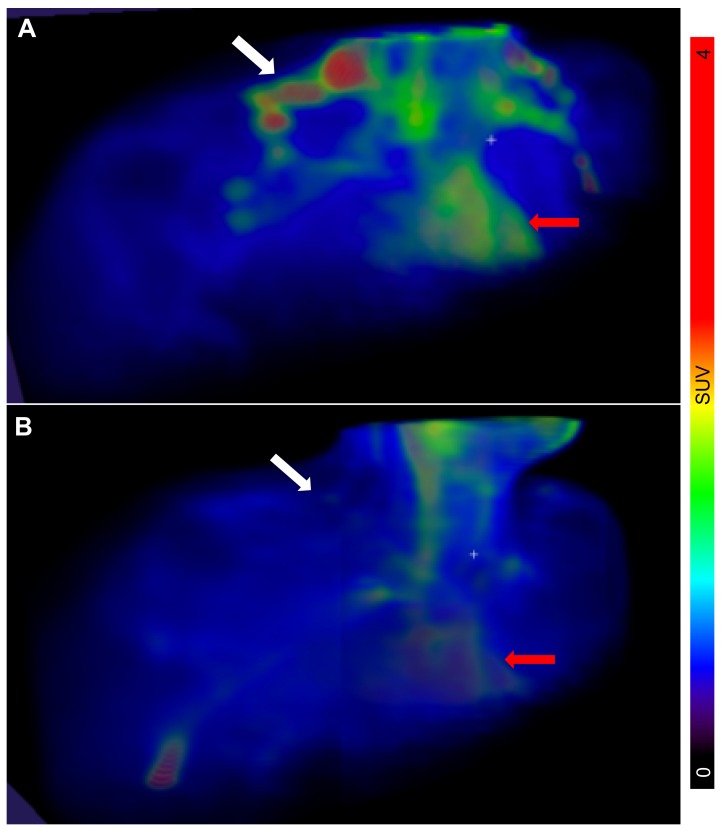
3D maximum intensity projections of the biodistribution of 2-deoxy-2-(^18^F)fluoro-D-glucose (^18^F-FDG) in the neck region, following cold stimulation. The location of the supraclavicular regions where brown adipose tissue (BAT) is usually located is indicated by white arrows. Heart is indicated by red arrows. There was a marked intersubject variability in ^18^F-FDG uptake, i.e., activated BAT, in the enrolled individuals with type 1 diabetes (T1D), ranging from very high (**A**) to low or absent (**B**). Representative images are shown. The scale bar to the right shows the colour coding from SUV 0 to 4.

**Figure 2:**
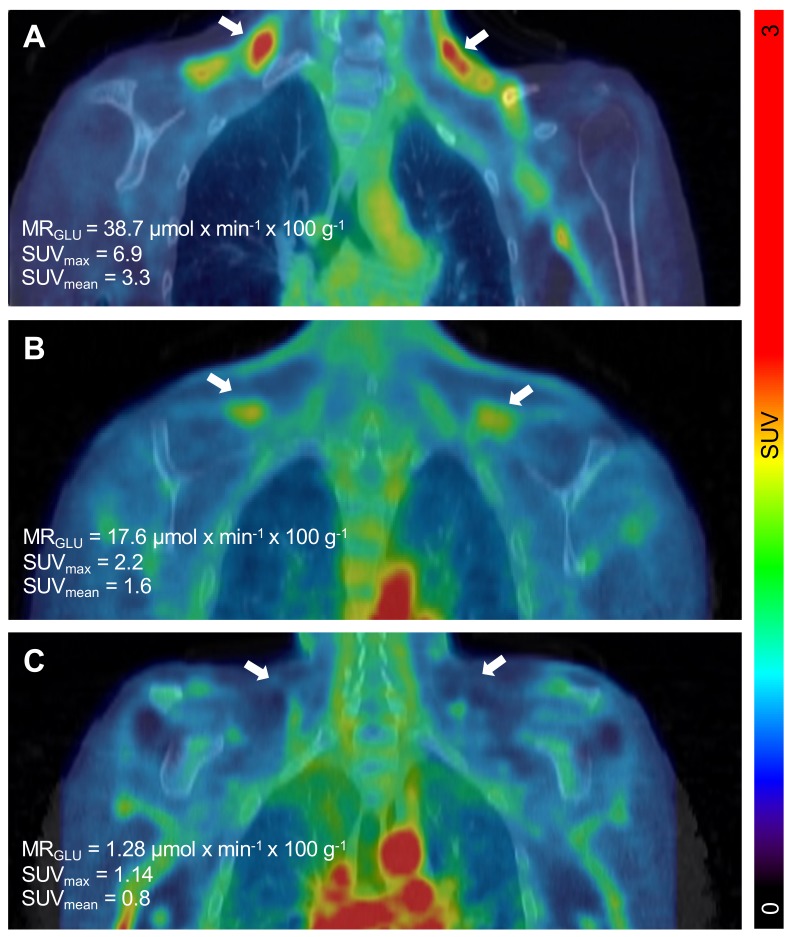
Detection of a wide range of metabolically active brown adipose tissue (BAT) in subjects with T1D. Cold-induced metabolic activity of BAT was measured by dynamic ^18^F-FDG PET/CT individuals with type 1 diabetes. Representative coronal PET/CT image projections of individuals with high (38.7 µmol × min^−1^ × 100 g^−1^) (**A**), intermediate (17.6 µmol × min^−1^ × 100 g^−1^) (**B**), or low (µmol × min^−1^ × 100 g^−1^) (**C**) amounts of metabolically active BAT deposits. Left and right deposit of BAT is indicated by white arrows in all images. The scale bar to the right shows the colour coding from SUV 0 to 3.

The PET/CT examinations in all *n* = 11 subjects were successful. Descriptive data of all participants can be found in Table 1. The BAT metabolic rate of glucose per grams of tissue (MR_Glu_) varied substantially between individuals, ranging from 0.75 µmol × min^−1^ × 100 g^−1^ (indicating negligible BAT activity) to 38.7 µmol × min^−1^ × 100 g^−1^ (Figure 1 and Figure 2).

**Figure 3:**
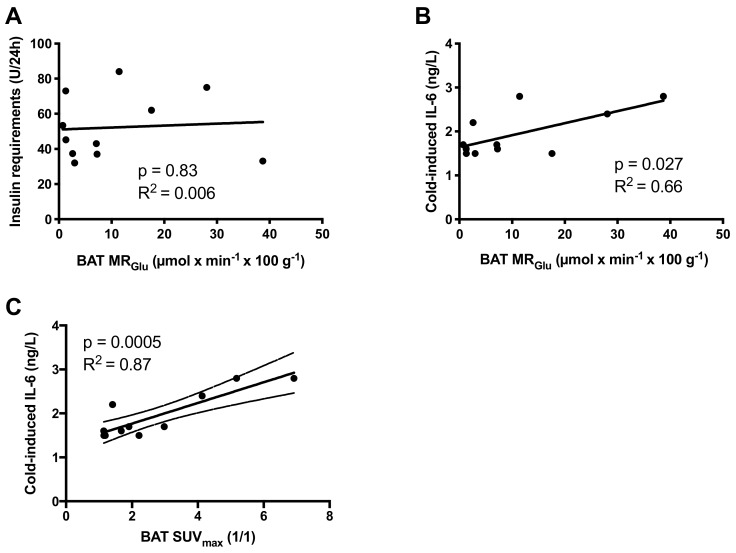
There was no correlation between the brown adipose tissue (BAT) activity as assessed by MR_Glu_ and the daily insulin requirements in individuals with T1D (**A**). The plasma levels of potential batokine IL-6 correlated with levels of functional BAT assessed as either MR_Glu_ (**B**) and SUV_max_ (**C**).

We found no correlation between metabolic activity of BAT and insulin requirements, whether expressed as total daily insulin doses (*p* = 0.83), weight adjusted (*p* = 0.64), or adjusted for lean body mass (*p* = 0.67) (Figure 3A). The metabolic activity of BAT did not correlate to fasting blood glucose (*p* = 0.91) or HbA1c (*p* = 0.99). BAT activity was not correlated to blood lipid profiles: total cholesterol (*p* = 0.72), HDL-cholesterol (*p* = 0.86), LDL-cholesterol (*p* = 0.58) or triglycerides (*p* = 0.78), nor did the metabolic activity of BAT correlate to body composition expressed as body mass (*p* = 0.71), BMI (kg/m^2^, *p* = 0.72), fat mass (*p* = 0.93), or lean body mass (*p* = 0.71).

However, the metabolic activity of BAT correlated to the cold-induced levels of IL-6 (*r* = 0.44, *p* = 0.027) (Figure 3B). Cold-induced levels of IL-6 similarly correlated to BAT activity as assessed by maximal Standardized Uptake Values (SUV_max_) (*r* = 0.87, *p* = 0.0005) (Figure 3C) or mean Standardized Uptake Values (SUV_mean_) (*r* = 0.86, *p* = 0.0006).

The cold-induced levels of IL-6 were not, however correlated to fasting glucose, HbA1c, or insulin requirements (data not shown), whereas the IL-6 levels prior to cooling (*r* = 0.80, *p* = 0.003) and the delta IL-6 levels (*r* = 0.85, *p* = 0.0009) correlated positively to HbA1c levels but not insulin requirements or fasting glucose. There was a tendency towards a positive correlation to IGF-1 levels both prior to cooling (*r* = 0.58, *p* = 0.064) and cold-induced (*r* = 0.54, *p* = 0.083). No correlation was observed for FGF-21 (*p* = 0.47), leptin (*p* = 0.70), or adiponectin (*p* = 0.60).

The metabolic activity of WAT was found to correlate with fasting glucose levels (*r* = 0.73, *p* = 0.01) but not with HbA1c (*p* = 0.49). However, metabolic activity of WAT did not correlate to insulin requirements, markers of body composition, or investigated hormones/cytokines. The metabolic activity of WAT and skeletal muscle were correlated (*r* = 0.89, *p* = 0.0002).

As for BAT, we did not observe any correlation between the metabolic activity in skeletal muscle and insulin needs, fasting glucose, HbA1c, or investigated hormones/cytokines. However, there was a negative correlation with body mass (*r* = −0.64, *p* = 0.036) and body volume (*r* = −0.63, *p* = 0.037), but not with fat mass (*p* = 0.27) or lean body mass (*p* = 0.16).

Notably, four participants had markedly increased BAT activity (23.94 ± 6.0 vs. 3.32 ± 1.03 µmol × min^−1^ × 100 g^−1^, *p* = 0.001). These four individuals did not differ when compared to the individuals with low BAT activity regarding markers of metabolic control or body composition (data not shown). However, there was a tendency towards increased insulin requirements in these four patients (0.79 ± 0.11 vs. 0.58 ± 0.03 U × kg^−1^ × 24 h^−1^, *p* = 0.053). In addition, the cold-induced levels of IL-6 were significantly higher in the patients with high BAT activity (2.4 ± 0.31 vs. 1.7 ± 0.09 ng/l, *p* = 0.024). Both the pre-cooling and cold-induced levels of FGF-21 were increased in the patients with high BAT activity (pre-cooling 185 ± 79 vs. 44 ± 12 pg/mL, *p* = 0.04 and cold-induced 144 ± 51 vs. 40 ± 12 pg/mL, *p* = 0.03).

**Figure 4:**
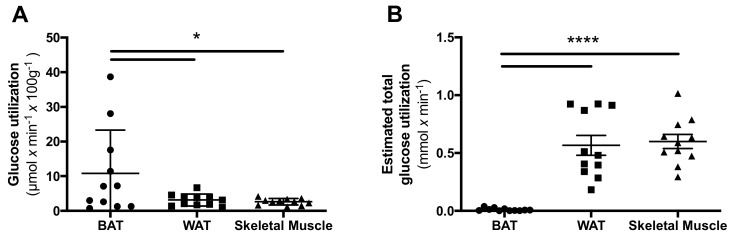
Glucose utilization in different important tissue compartments. The glucose utilization per gram tissue was found to be highest in brown adipose tissue (BAT), compared to white adipose tissue (WAT) and skeletal muscle (**A**). However, when estimating the total body contribution of glucose utilization in each tissue the contribution of BAT was minimal (**B**). * denotes *p* < 0.05, and **** *p* < 0.0001.

The glucose utilization per gram tissue was higher in BAT when compared both to WAT (*p* = 0.049) and to skeletal muscle (*p* = 0.039) (Figure 4A). However, when estimating the total amount of tissue in each compartment, the total glucose utilization towards whole body metabolism was significantly higher in both skeletal muscle (*p* < 0.0001) and WAT (*p* = 0.0001) when compared with BAT (Figure 4B).

## 3. Discussion

We demonstrated, for the first time, the presence of BAT in individuals with T1D. The metabolic activity of BAT ranged from considerable in some individuals (>10 µmol × min^−1^ × 100 g^−1^) to negligible in others. Using SUV_mean_ > 1.5 as defining cut-off for the presence of functional BAT [13], we reported a prevalence of 36% (4/11) in this study on individuals with T1D. This is in line with the prevalence in non-diabetic subjects using similar methodologies. However, an additional three individuals exhibited BAT SUV_mean_ between 1.2 and 1.5, just below the threshold. SUV_max_ is usually not used for assessing BAT prevalence as it potentially is less reproducible than SUV_mean_ in this specific setting [13]. Regardless, the prevalence of BAT in individuals with T1D was also 36% (4/11) when using SUV_max_ > 2.5 as threshold.

BAT contributes to non-shivering thermogenesis via its oxidative metabolism [14] but can also, at least in animals, clear glucose from the circulation via its high metabolism [15,16]. This could, at least theoretically, serve as a protective mechanism in order to counteract hyperglycaemia. In the present study, we found that the glucose utilization per gram tissue was significantly higher in BAT compared to both WAT and skeletal muscle. However, when approximating the total amount of tissue in the body, both skeletal muscle and WAT consumed more glucose in total than BAT. A limitation of this study is the potentially poor accuracy of the muscle mass approximation, as it was estimated based on a population average assumption of muscle mass percentage. Despite this, the general conclusion should be reliable as the difference was very large.

However, BAT can secrete hormones and cytokines which exert a systemic effect and can thereby impact metabolic control and energy expenditure [11].

We found no correlation between the metabolic activity of BAT and exogenous insulin requirements or markers of metabolic control (HbA1c and fasting glucose) in patients with T1D. There was no difference in the metabolic activity of BAT in participants with low insulin requirements compared to those with high insulin requirements. Nor were insulin requirements lower in the four participants with the highest metabolic activity of BAT. In fact, there was a tendency towards increased insulin requirements in the individuals with the highest metabolic activity of BAT.

However, the metabolic activity of BAT correlated with the levels of IL-6, a known batokine, and was found to be higher in participants with the highest BAT activity. This is suggestive of a systemic effect of BAT in T1D. Traditionally, IL-6 is viewed as a pro-inflammatory cytokine, but it can also exert endocrine effects and has been described to increase insulin sensitivity in both skeletal muscle and WAT [1,2]. In addition, IL-6 has been shown to be necessary for the browning of WAT in experiments using IL-6-null mice [17]. However, high levels of IL-6 have also been linked to insulin resistance [3]. In addition to the diverse role of IL-6 in metabolic control, another dimension of complexity is introduced in T1D, since IL-6 has also been ascribed to play a role in this pathogenesis [18]. We observed that the cold-induced levels of IL-6 correlated with metabolic activity of BAT, which was suggestive of an increased secretion of IL-6 from BAT upon cold exposure. However, the cold-induced levels of IL-6 were not correlated with markers of metabolic control, whereas the levels of IL-6 prior to cooling and the delta IL-6 levels were positively correlated with HbA1c. Whether this finding represented a causal correlation of IL-6 contributing to increased glycaemia or a compensatory effect of IL-6 aiming to increase insulin sensitivity in T1D remains to be determined.

In the publication by Gunawardana et al., diabetic mice were cured by a transplantation of embryonic BAT [4,5]. The levels of adiponectin, leptin, and IGF-1 were found to be increased post-transplantation [4]. The insulin levels were statically low and comparable to that of untreated diabetic mice, suggesting an insulin-independent mechanism for the restoration of glycaemic control [4]. However, when an insulin receptor antagonist (S-961) was injected, the transplanted mice failed to restore normoglycaemia. The glucose-lowering effect was therefore suggested to be mediated by IGF-1 [4]. IGF-1 and insulin have separate receptors but can exert overlapping effects, although the affinity of IGF-1 to the insulin receptor is only 1:100 compared to insulin [19]. IGF-1 has been reported to be reduced in adults, adolescents, and children with T1D [20,21,22]. In fact, it has previously been shown that subcutaneous administration of IGF-1 reduces insulin needs in patients with T1D [23,24]. It has also been shown in rats that cold exposure increases the expression of IGF-1 in BAT, and that plasma from rats exposed to cold increases both the proliferation rate of brown adipocyte precursor cells and the expression of IGF-1 in vitro [25]. We found a tendency towards a positive correlation between the metabolic activity of BAT and plasma levels of IGF-1, but no correlation between metabolic activity of BAT and the levels of leptin and adiponectin. This might be due to species differences or differences in embryonic and adult BAT. It is also possible that individuals with a high metabolic activity of BAT have higher levels of IGF-1 locally in BAT, which are not reflected by an increase in circulating levels of IGF-1.

FGF-21 was recently shown to correlate to the metabolic activity of BAT in healthy individuals [26]. FGF-21 has been shown to improve insulin sensitivity, reduce blood glucose levels, and preserved beta-cell function in mice [27]. We did not observe a direct correlation between BAT activity and plasma levels of FGF-21. However, we found that the levels of FGF-21 were increased in the patients with the highest BAT activity. At least in part, these differences could be ascribed to the reduced levels of FGF-21 and altered secretion regulation observed in T1D [28].

We identified four individuals with functional BAT, defined as SUV_mean_ > 1.5 [13]. These individuals exhibited a markedly increased BAT glucose utilization, with a MR_Glu_ ranging from 11.4 to 38.7 (average 23.94 ± 6.0) µmol × min^−1^ × 100 g^−1^. Despite that, we did not observe any signs of improved metabolic control in those individuals. It is tempting, based on preclinical literature, to speculate that BAT could serve as a back-up mechanism in order to clear glucose from circulation and thereby protect against severe hyperglycaemia in T1D under hypoinsulinemic conditions. However, we found no evidence for such a mechanism under these experimental conditions.

The major limitation of this study was the low number of enrolled individuals (*n* = 11). The original primary endpoint was to evaluate whether BAT glucose utilization correlated with insulin requirement in subjects with T1D. At the interim analysis at *n* = 11 individuals, no such tendency was observed, as outlined above. Further enrollment was thus deemed unnecessary. The remaining correlations observed in this material should therefore be considered exploratory, as the study was not specifically designed or powered otherwise. We hope, however, that the exploratory observations will generate novel hypotheses on the role of BAT in T1D and motivate future clinical studies. One such research question is the prevalence of functional BAT during the progress of T1D, which could be examined by a longitudinal study design following individuals from T1D debut.

The notion of the superclavicular deposits of functional BAT acting as a glucose sink in response to hyperglycemia seems unlikely to hold in humans. Generally, these deposits exhibited substantially higher glucose uptake than WAT or skeletal muscle in individuals with T1D on a per gram basis. However, given the relatively small amount of superclavicular BAT (approximately 100 g or less), the total body contribution to glucose clearance was negligible compared to that of the whole-body mass, especially of skeletal muscle, but also of WAT. It remains to be investigated whether induced browning of adipose tissue might increase the total beige/brown adipose tissue to meaningful levels, which might be able to help correct hyperglycemia in humans.

## 4. Materials and Methods

The study was approved by the regional ethical board of Uppsala County (2015/191, approval date 2015-06-10) and conducted in accordance with the declaration of Helsinki as revised in 2000. Participants were recruited from the Diabetes and Endocrinology Department at Uppsala University Hospital. All study participants were given oral and written information and gave their written consent prior to participation in the study. Inclusion criteria were: clinically established diagnosis of T1D, age 18–35 years, BMI 19–25 kg/m^2^, HbA1c 45–60 mmol/mol (DCCT 6.3–7.6%). Exclusion criteria were: other metabolic disorders, treatment with glucose-lowering drugs other than insulin, drug abuse, extreme physical activity, and pregnancy. In total, 11 individuals with T1D were included. Participants were sub-grouped based on their average insulin requirements: low (<0.65 U × kg^−1^ × 24 h^−1^) or high insulin needs (>0.65 U × kg^−1^ × 24 h^−1^), see Table 1 for descriptive data of all participants. Insulin doses were based on self-recorded data over a five-day period, and a daily average was calculated. Blood samples were collected at inclusion and again on the day of the PET/CT-examination. Plasma was separated from EDTA tubes and immediately frozen and stored in −80 °C. The study is registered at clinicaltrials.gov, identifier NCT02984514.

### 4.1. Metabolic Parameters

Fibroblast growth factor-21 (FGF-21), leptin, and adiponectin were analyzed using commercially available ELISA kits (Art. No *DF2100*, *DLP00* and *DRP300* respectively, R&D Systems, Minneapolis, MN, USA) according to the manufacturer’s protocol. All other blood samples were analyzed at the central laboratory of Uppsala University Hospital according to clinical routine.

### 4.2. Body Composition

All examinations were conducted in the morning under fasting conditions. Body weight and volume was determined by air displacement plethysmography (Bod Pod) (Life Measurements Inc., Concord, CA, USA) and body density was calculated based on these two measurements. Body fat mass and lean body mass were determined by a two-compartment model. Measurements of waist, hip, and upper arm circumference were taken by the same examiner for all patients according to standardized techniques.

### 4.3. Assessment of Tissue Glucose Utilization by ^18^F-FDG PET/CT Examinations

All PET/CT examinations were conducted under fasting conditions. Blood samples were collected prior to cooling and after cooling just prior to the PET/CT examination. To induce BAT activation, participants rested in light clothing in a room with an ambient temperature of 19 °C for 60 min while wearing a cooling vest (Irsus AB, Lund, Sweden).

Participants were positioned in a Discovery ST PET/CT scanner (GE Healthcare, Chicago, IL, USA) centred at the clavicular level. A localization dose CT examination (140 kV, Auto mA 10–80 mA) was performed for attenuation and anatomical co-registration of PET images. A target dose of 2 MBq/kg ^18^F-FDG was administered intravenously and each participant was examined by dynamic PET for 40 min.

The PET list data was sorted into 11 frames (4 × 30 s, 1 × 60 s, 1 × 120 s, 3 × 300 s, 2 × 600 s) and reconstructed using OSEM VUEPOINT (2 iterations/21 subsets, Matrix 128 × 128 pixels, post filter 6 mm, 50 cm diameter zoom).

Regions of interest (ROIs) were placed in the supraclavicular regions where BAT is normally located, guided by CT (−190 to −10 Houndsfield units) according to standard practise [13]. ROIs were also segmented in WAT deposits (posterior neck adipose tissue) in skeletal muscle (erector spinae). R Voxels fully within the lumen of the aortic arch were also identified and used as the dynamic blood pool input function for kinetic modeling of tissue PET uptake.

The metabolic rate of glucose per grams of tissue (MR_Glu_, µmol × min^−1^ × 100 g^−1^) in BAT (Lumped Constant (LC) 1.15), WAT (LC 1.0) and skeletal muscle (LC 1.2) was calculated from dynamic PET data by Patlak graphical analysis using the signal from the aortic arch as arterial blood input and the average B-glucose over the scan for normalization (PKIN module, PMOD 3.4, PMOD Technologies, Zurich, Switzerland). The mean standardized uptake values (SUV_mean_) and maximal standardized uptake values (SUV_max_) at the 40 min time-point were also calculated for each region.

The total glucose utilization (µmol × min^−1^) in each tissue was approximated by multiplying the MR_Glu_ per grams of tissue (µmol × min^−1^ × 100 g^−1^) by the estimated total mass of BAT (by direct segmentation on PET/CT images), WAT (BodPod), and skeletal muscle (estimated from individual body weight multiplied by the population-based muscle percentage in males (35%) and females (25%).

### 4.4. Statistical Analysis

Statistical analysis was performed using Graph Pad Prism version 6.03 (GraphPad Software Inc., San Diego, CA, USA). Two-tailed Student’s *t* test was used to compare differences between two groups. The presence of autoantibodies was compared using Fisher’s exact test. Comparisons between more than two groups were performed using a one-way ANOVA with Tukey’s post-hoc test. Normality distribution was determined using D´Agostino–Pearson omnibus normality test. All data used for statistical analysis passed the normality test. Correlations were therefore calculated by linear regression using Pearson’s product moment correlation. Values are given as means ± SEM, *p*-values < 0.05 were considered statistically significant.

## 5. Conclusions

Cold-induced BAT was detected in patients with T1D with a varying degree of metabolic activity. The metabolic activity of BAT was correlated to the cold-induced levels of IL-6. However, the normal physiological range of metabolic activity in BAT had no impact on insulin requirements and metabolic control in T1D.

## Figures and Tables

**Table 1:** Descriptive data of individuals with type 1 diabetes with either low or high insulin needs.

Parameter	Low Insulin Needs	High Insulin Needs	*p*-Value
Total number	6	5	-
Male/female	3/3	4/1	-
Age (years)	29 ± 2.2	32.2 ± 1.5	0.28
Age at disease onset (years)	12.3 ± 3.2	16 ± 2.8	0.42
Disease duration (years)	16.7 ± 2.4	16.4 ± 1.9	0.94
Body Mass Index (BMI) (kg/m^2^)	23.4 ± 1.1	25.6 ± 0.7	0.13
Fat mass (kg)	15.1 ± 3.4	22.4 ± 1.6	0.10
Fat-free mass (kg)	56.3 ± 3.8	61.8 ± 5.5	0.35
HbA1c (mmol/mol, NGSP%)	54 ± 2.4 (7.1 ± 0.2)	58 ± 3.3 (7.5 ± 0.3)	0.37
Fasting glucose (mmol/L)	9.7 ± 1.2	10.4 ± 2.2	0.76
Fasting C-peptide* (nmol/L)	0.067 ± 0.04	0.003 ± 0.0	0.18
GAD-65 positive (%)	66	80	1.0
IA-2 positive (%)	33	60	0.57
Total insulin doses (U × 24 h^−1^)	37.9 ± 2.1	69.5 ± 5.3	0.0002
Weight adjusted insulin doses (U × 24 h^−1^ × kg^−1^)	0.53 ± 0.02	0.82 ± 0.05	0.0002
Brown adipose tissue (BAT) glucose utilization (µmol × min^−1^ × 100 g^−1^)	10 ± 5.8	11.8 ± 5.1	0.82
White adipose tissue (WAT) glucose utilization (µmol × min^−1^ × 100 g^−1^)	3.4 ± 0.5	2.8 ± 1	0.64
Skeletal muscle glucose utilization (µmol × min^−1^ × 100 g^−1^)	2.9 ± 0.28	2.3 ± 0.5	0.37
IL-6 (ng/L)	1.9 ± 0.2	2 ± 0.3	0.73
IGF-1 (µg/L)	184 ± 26	167 ± 12	0.59
FGF-21 (ng/L)	41 ± 14	122 ± 45	0.0951
Adiponectin (µg/L)	12879 ± 3416	8399 ± 1959	0.31
Leptin (ng/L)	9229 ± 4120	8763 ± 2044	0.93

* C-peptide could only be detected in three patients and the remaining patients was therefore ascribed the lowest detectable level of the clinical assay (0.003 nmol/l). Data on GAD-65 and IA2 are presented as percentage of positive patients using a cut-off value > 5 IE/mL for GAD-65 and > 8 kE/L for IA-2. All other values are presented as mean ± SEM.
